# Counting loops in sidechain-crosslinked polymers from elastic solids to single-chain nanoparticles†
†Electronic supplementary information (ESI) available: Synthetic procedures, methods and materials, and characterization data; Monte Carlo simulation algorithm for obtaining *φ*_*λ*_ for side-chain crosslinked polymer networks. See DOI: 10.1039/c9sc01297d


**DOI:** 10.1039/c9sc01297d

**Published:** 2019-05-01

**Authors:** Junpeng Wang, Rui Wang, Yuwei Gu, Alexandra Sourakov, Bradley D. Olsen, Jeremiah A. Johnson

**Affiliations:** a Department of Chemistry , Massachusetts Institute of Technology , Cambridge , MA 02139 , USA . Email: jaj2109@mit.edu; b Department of Chemical Engineering , Massachusetts Institute of Technology , Cambridge , MA 02139 , USA . Email: bdolsen@mit.edu

## Abstract

The vast differences in material properties accessible *via* crosslinking of sidechain-functionalized polymers are driven by topology. For example, vulcanized rubbery networks feature intermolecular connections and loop topologies of various orders while single-chain nanoparticles (SCNPs) are comprised, in principle, entirely of primary loops. Despite this fact, precise quantification of loops in sidechain crosslinked polymers has not been accomplished. Here, it is demonstrated that by introducing cleavable linkers and mass labels onto the pendant functional groups of reactive polymers, the number of primary loops in sidechain crosslinked materials ranging from rubbery networks (gels) to soluble SCNPs can be precisely quantified. This study sheds new light on the topology of sidechain-crosslinked networks, providing design principles for augmenting the properties of this industrially and academically important class of materials through topological control.

## Abstract 2

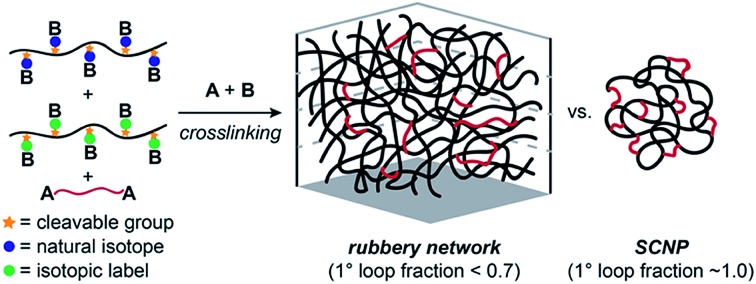
The vast differences in material properties accessible *via* crosslinking of sidechain-functionalized polymers are driven by topology.

## 

Crosslinked polymers are widely used in applications including gas storage and separation,^1^–^4^ water purification,^5^–^7^ soft robotics^8^,^9^ and additive manufacturing.^10^–^12^ Sidechain-crosslinked networks made from reactive polymers with f functional groups (**B_f_**) and crosslinkers (**A_2_**) represent a particularly useful class of polymer networks (Fig. 1a). Perhaps the most famous example is vulcanized rubber, which is formed by crosslinking of polyisoprene natural rubber with sulphur.^13^ Single-chain nanoparticles (SCNPs) are another type of side-chain crosslinked polymer that have attracted extensive attention.^14^–^18^ Notably, the difference between vulcanized rubber, which is an elastic solid that is insoluble in solvents, and SCNPs, which are nano-scale soluble materials, is one of topology:^19^ vulcanized rubber is formed *via* mostly intermolecular reactions (Fig. 1b), while SCNPs are, in principle, comprised of only primary (1°) loops (*i.e.*, “self-crosslinking” or “internal crosslinking”^20^ where both ends of the **A_2_** crosslinker are connected to the same **B_f_** macromolecule, Fig. 1b). Thus, it is important to quantify and control the topology of sidechain-crosslinked polymers to fully understand their structure and engender novel properties for emerging applications.^21^

**Fig. 1:**
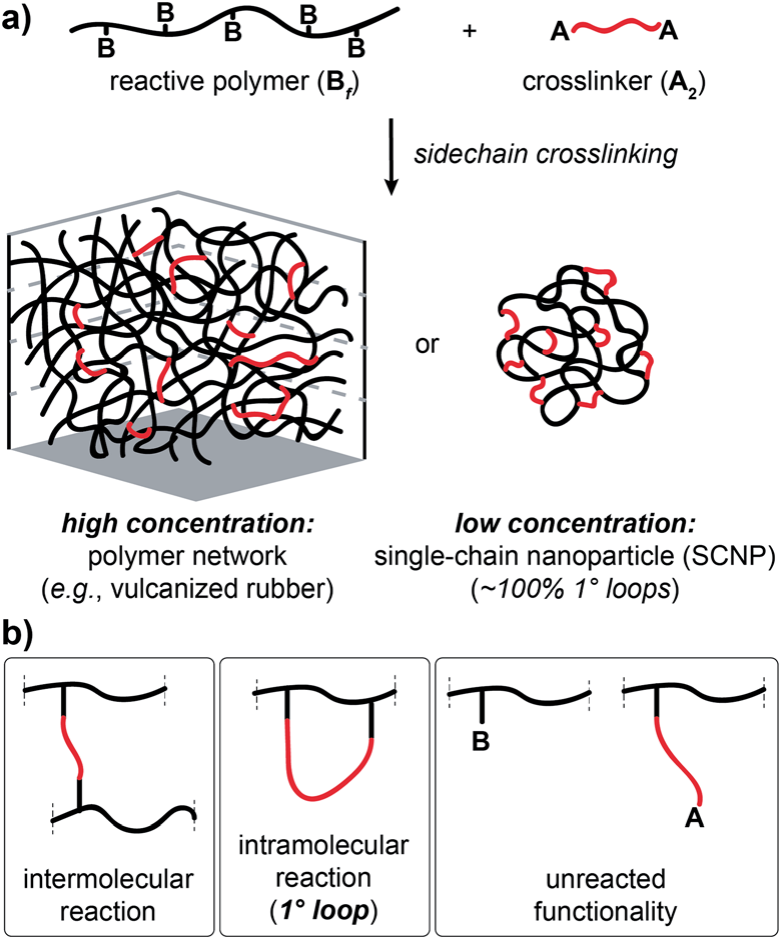
(a) Schematic for sidechain crosslinking of polymer **B_f_** with crosslinker **A_2_**. By varying topology, one can access a wide range of material properties from elastic networks to SCNPs. (b) Molecular features of sidechain crosslinked polymers.

Network disassembly spectrometry (NDS) is a powerful tool for precisely counting loops of various orders in end-linked polymer networks.^22^–^26^ NDS is based on the concept of a crossover experiment, wherein mass-labeled precursors are used to construct networks where the mass labels will distribute uniquely at loops of a specific order compared to all other network topologies. Chemical degradation of the networks and mass spectrometry of the labeled fragments provides the loop fractions. Compared to other methods for quantifying polymer network topology,^27^–^29^ NDS can selectively and unambiguously quantify loops of a specific order. Nevertheless, due to the requirement for mass-labeled components, NDS has so far only been used in the context of end-linked model networks; developing an analogous approach for studying the topology of sidechain-crosslinked polymers would provide new insights into the structure of these ubiquitous materials. In particular, NDS for such networks would provide a fundamentally new way to quantify the topological purity of SCNPs, which hitherto has been inferred by measurement of macromolecular size.

Here, we present an NDS approach for counting 1° loops in sidechain-crosslinked polymers (Fig. 2). By introducing cleavable groups and mass labels adjacent to the pendant functional groups of reactive polymers **B_fH_** and **B_fD_** (Fig. 2a), the 1° loop fraction, *φ*_*λ*_, in networks derived from crosslinking a mixture of 2*x***B_fH_** and 2(1 – *x*) **B_fD_** with bifunctional crosslinker **A_2_** can be obtained from the mass distribution of the degradation products **nn**, **ni**, and **ii** (Fig. 2b) *via*eqn (1) and (2).1
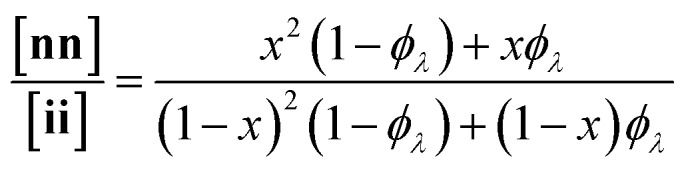

2
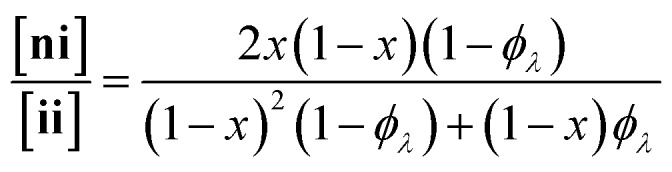



**Fig. 2:**
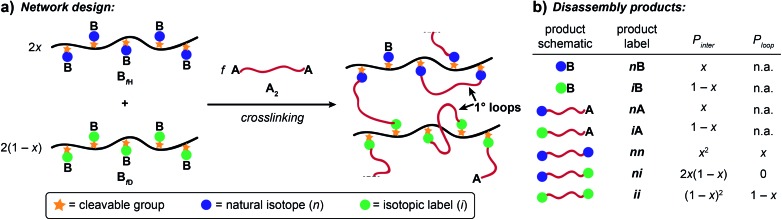
Strategy for counting 1° loops in sidechain-crosslinked polymer networks. (a) Polymers with cleavable groups and mass labels on pendant functional groups (**B_fH_** and **B_fD_**) are mixed together and crosslinked with **A_2_**, yielding a degradable network. (b) Degradation products obtained from such networks. Unreacted functionalities yield **nB** or **iB** and **nA** or **iA** products. Fully reacted **A_2_** generate three possible labeled products: **nn**, **ni**, and **ii**. The probabilities of forming each product at intermolecular *versus* 1° loop junctions are given as *P*_inter_ and *P*_loop_, respectively. The ratios of **nn** : **ni** : **ii** vary as a function of the primary loop fraction, *φ*_*λ*_.

It is shown that *φ*_*λ*_ in sidechain-crosslinked rubbery networks can be exceptionally high; due to the high functionality of **B_f_**, these networks are “loop defect tolerant” similar to other high branch functionality networks.^30^,^31^ Moreover, as for end-linked networks,^21^,^26^ *φ*_*λ*_ plays a major role in determining the shear storage modulus (*G*′) of sidechain-crosslinked rubbery networks. At high dilution, soluble networks with *φ*_*λ*_ > 0.7 are obtained. Notably, even at very low concentrations (<1 mg mL^–1^ of **B_f_**) that are often used for the synthesis of SCNPs, NDS reveals the presence of a small fraction (∼3–7%) of intermolecular reactions that strongly depends on the length of **A_2_**. Based on these results, NDS should be applied broadly to elucidate the topological purity of SCNPs.

To demonstrate the concepts outlined in Fig. 2, nitroxide-mediated polymerization of styrene and 4-vinylbenzyl acetate (5.5 : 1 ratio) was conducted to provide random copolymer^32^,^33^ **1** (*M*_n_ = 14 600, *Đ* = 1.16, Fig. 3a), which was hydrolyzed to form hydroxyl-functionalized copolymer **2**. Coupling **2** with acids **3H** or **3D**, which contain either C_4_H_8_ or C_4_D_8_ labels, respectively, and an azide end group, yielded copolymers **B_20H_** and **B_20D_** with mass labels and azide pendant functional groups (Fig. 3a) (*M*_n_ = 17 300, *Đ* = 1.17, average number of azide groups per polymer = 20, see ESI† for details). **B_20H_** and **B_20D_** were combined in a 1 : 1 ratio, and this mixture was used to generate two sets of sidechain crosslinked polymer networks from **A_2_** crosslinkers of different length (PEG4 and PEG12, Fig. 3b) *via* copper-catalyzed azide–alkyne cycloaddition (CuAAC).^34^–^39^ The resulting materials were hydrolyzed, and the degradation products (Fig. 3b, far right) were analyzed by LC/MS (Fig. 3c and d). Conversion was >95% for all samples (unreacted functionalities will appear as LC/MS peaks with unique masses, see Fig. 2b, allowing their facile quantification). *φ*_*λ*_ values for networks derived from both PEG4 and PEG12 **A_2_** at different concentrations were obtained (Fig. 3e) from eqn (1) and (2). Similar to end-linked networks,^23^ the tendency to form loops in these sidechain crosslinked materials strongly depends on **A_2_** length: networks prepared from the longer PEG12 **A_2_** consistently had fewer loops than those prepared from PEG4 **A_2_**. In both sets of materials, the maximum *φ*_*λ*_ for which gelation could occur was ∼0.7; above this value, soluble materials were obtained. Based on Monte Carlo simulations, it was previously reported that gelation can occur in tetrafunctional end-linked networks (**A_2_** + **B_4_**) when *φ*_*λ*_ < ∼0.35.^40^ Given that the maximum *φ*_*λ*_ for gelation increases with the junction functionality (*n* in this study), the observation of a critical *φ*_*λ*_ of ∼0.7 for gelation suggests that the effective functionality of **B_f_** is much larger than 4, which is consistent with its chemical structure (*n* = 20). The ability to form robust rubbery networks even with quite high 1° loop densities, *i.e.*, “loop defect tolerance,” is a hallmark of high branch functionality polymer networks.^30^,^31^

**Fig. 3:**
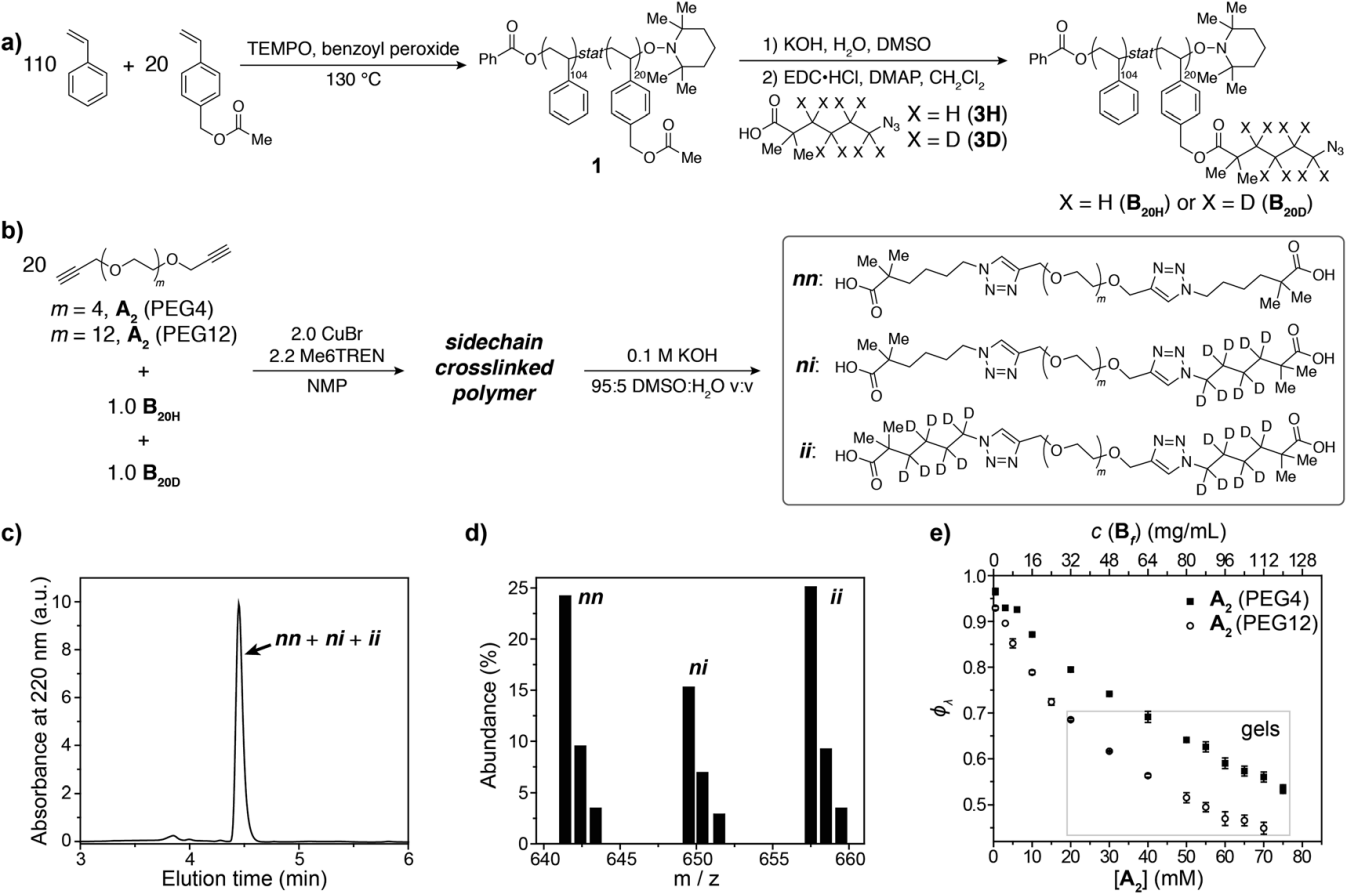
(a) Synthesis of azide-functionalized statistical copolymers **B_20H_** and **B_20D_**. (b) Synthesis and degradation of sidechain-crosslinked polymer networks derived from **B_20H_** and **B_20D_** and **A_2_** (PEG4 or PEG12) crosslinkers provides three different doubly reacted products **nn**, **ni**, **ii** that vary by their isotopic labeling pattern. (c) Absorbance (220 nm) *vs.* elution time LC trace for degraded network formed from **A_2_** PEG4. The major peak corresponds to the mixture of products **nn**, **ni**, and **ii**. (d) Representative mass spectrum showing the products **nn**, **ni**, and **ii**. The ratios of these products are dependent on *φ*_*λ*_*via*eqn (1) and (2). (e) Measured *φ*_*λ*_ for networks derived from **A_2_** PEG4 and PEG12 at different concentrations. Gels are formed when *φ*_*λ*_ < 0.7 and soluble networks are formed when *φ*_*λ*_ > 0.7. The material formed from **A_2_** PEG4 at 0.8 mg mL^–1^ of polymer has *φ*_*λ*_ = 0.97 ± 0.01.

When *φ*_*λ*_ > ∼0.7 in these materials, the preponderance of 1° loops precludes the gel point and soluble networks are obtained. Depending on the polymer mass concentration (*c*), a wide range of *φ*_*λ*_ values were observed. When *c* < 5 mg mL^–1^, *φ*_*λ*_ was >0.9. As described above, SCNPs are defined as sidechain-crosslinked polymers with 100% 1° loops. Previous studies by Kuhn and Balmer on crosslinking of polyvinyl alcohol and terephthalaldehyde suggested that 1° loop formation dominated when the polymer concentration was ∼1 mg mL^–1^ or lower.^20^ Moreover, SCNPs are typically formed at *c* ∼ 1 mg mL^–1^.^16^ Here, in the PEG4 and PEG12 materials formed at 0.8 mg mL^–1^, *φ*_*λ*_ was 0.97 ± 0.01 and 0.93 ± 0.01, respectively. Thus, even at *c* < 1 mg mL^–1^ the 1° loop fraction is not 100%. In addition, these results demonstrate that the formation of SCNPs with 100% topological purity becomes significantly more challenging as the crosslinker (here, **A_2_**) becomes larger.

When the statistics of functional group placements are appropriately captured, the experimentally measured *φ*_*λ*_ is qualitatively reproduced by Monte Carlo simulations without any adjustable parameters (see ESI section† for a detailed description of the MC algorithm). To explore how the distribution of groups on **B_f_** affects 1° loop formation, three conditions were explored: point crosslinkers (*centralized*), where **B_f_** is viewed as a point with 20 reactive groups (this approach is equivalent to previous studies using end-linked networks^41^), functional groups equally spaced by *d* monomers (*equal*), and randomly distributed functional groups (*random*). As shown in Fig. 4a and b, the *centralized* model significantly overestimated *φ*_*λ*_, which is not surprising since the close proximity of the reactive groups in this model should favor intramolecular reactions. In contrast, the *equal* model using *d* = 5 based on the DP of unfunctionalized PS = 100 and *n* = 20 underestimated *φ*_*λ*_ for high concentrations. Since the pendant functional groups are not evenly distributed along the statistical copolymers used experimentally, this discrepancy highlights the impact of randomness on *φ*_*λ*_, particularly overlooking pairs of reactive groups that randomly occur very close together on the polymer chain. This finding suggests that methods for precision polymerization, wherein reactive groups are precisely spaced along a polymer backbone, should provide a means for controlling the topology of sidechain-crosslinked networks and SCNPs.^42^–^47^ Finally, the *random* model, which describes **B_f_** as a perfectly random copolymer, gave the best agreement with experimental data, particularly for the PEG4 materials (Fig. 4a). There was still a discrepancy between experiment and simulations for PEG12 when [**A_2_**] > 30 mM or (*c* > 48 mg mL^–1^) (Fig. 4b). The extent of this discrepancy increased with polymer concentration, and it only occurred in the gelation regime, not in the soluble network regime. Kinetic and thermodynamic considerations not incorporated into the Monto Carlo simulations may explain these observations. Regarding the former, slower diffusion of the larger PEG12 **A_2_** crosslinker may hinder interchain crosslinking leading to an increase in *φ*_*λ*_ at high *c* values. This hypothesis was tested by lowering the catalyst loading for CuAAC from 1.0 equiv. to 0.2 equiv., which extended the gelation time by ∼5-fold. The *φ*_*λ*_ values for networks prepared under these two conditions were not significantly different (see ESI† for details), which is consistent with studies of end-linked networks showing that gelation kinetics have little effect on *φ*_*λ*_.^48^ Thus, diffusion limitations may not be the main cause of the observed variation between simulation and experiment in this work. An alternative explanation could be poor mixing between **A_2_** and **B_f_**, which may be amplified for the longer, more polar, **B_f_**. In the future, exploration of even longer **A_2_** crosslinkers and crosslinkers of different composition should provide further insights into this phenomenon; here, we note that without a direct measurement of *φ*_*λ*_ such discrepancies between simulation and experiment would be impossible to discern.

**Fig. 4:**
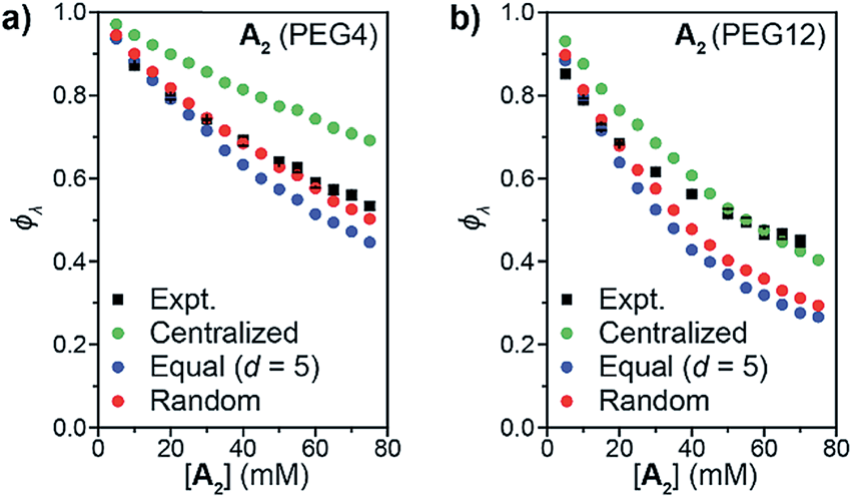
Comparison of predicted *φ*_*λ*_ from Monte Carlo simulation to experimental results (black squares) for **A_2_** PEG4 (a) and PEG12 (b). **B_f_** is treated using three different models: centralized functional groups (green circles), equal distance between functional groups (blue circles), and randomly distributed functional groups (red circles).

Oscillatory rheology was used to study the impact of 1° loops on *G*′ in these materials (Fig. 5a). In order to achieve the same *G*′, gels derived from PEG4 required higher concentrations compared to those derived from PEG12, which is consistent with the higher *φ*_*λ*_ and corresponding lower crosslinking density for the same concentration of PEG4 compared to PEG12. Normalizing *G*′ by 1 – *φ*_*λ*_ (Fig. 5b) provided a line with a slope of 910 J mol^–1^. According to eqn (3):

3 *G*′ = *Cν*_eff_*kT* = *Cν*_0_*kT*(1 – *φ*_*λ*_)

where *ν*_eff_ is the density of elastically effective strands, *ν*_0_ is the density of all strands, *k* is the Boltzmann constant, *T* is temperature, and *C* is a constant, if each reacted site is viewed as a trifunctional junction,^49^ then the observed slope (Fig. 5b) corresponds to a *C* value of 0.12, which is much lower than predicted for a trifunctional phantom network (*C* = 1/3) or an affine network (*C* = 1). Thus, even with knowledge of *φ*_*λ*_ it is not possible to predict *G*′ using classical theories, which support previous studies on end-linked networks that have demonstrated that 1° loops are not the only topological defects that impact *G*′; other topological features such as higher-order loops and network density fluctuations must also be considered for accurate prediction of *G*′.^21^,^25^,^26^,^47^ In addition, the statistically distributed nature of the functional groups in sidechain-crosslinked networks renders some strands too short to be elastically effective, causing a further decrease in *G*′. Establishing a theoretical model to predict *G*′ for sidechain-crosslinked networks is beyond the scope of this work, but the findings presented here suggest that primary loops, higher-order loops, as well as the distribution of pendant functional groups are parameters that should be considered.

**Fig. 5:**
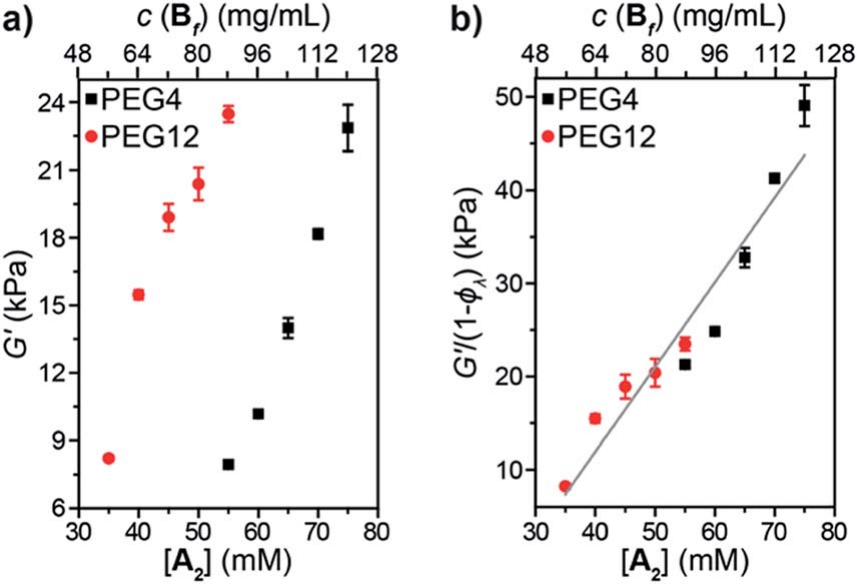
(a) Shear storage moduli (*G*′) *versus* concentration for **A_2_** PEG4 (black squares) and PEG12 (red circles) gels. (b) *G*′ normalized by *φ*_*λ*_*versus* concentration for **A_2_** PEG4 (black squares) and PEG12 (red circles) gels.

In summary, a conceptual framework that enables the precise quantification of 1° loops in sidechain-crosslinked polymers is presented and reduced to practice. Measurements of the primary loop fraction, *φ*_*λ*_, were made for networks derived from polymers bearing ∼20 reactive groups and two different crosslinkers of varied length (PEG12 and PEG28). In both sets of materials, gels could not form when the loop fraction is >∼0.7. Moreover, *φ*_*λ*_ was >0.9 when polymer concentrations of 5 mg mL^–1^ or lower were used. Notably, even at the lowest concentration tested (0.8 mg mL^–1^), which is within a range commonly used to form SCNPs, the largest value of *φ*_*λ*_ measured (for PEG4) was 0.97 ± 0.01, and this value was significantly lower for the samples derived from PEG12. These findings suggest that the topological purity of SCNPs is very sensitive to the size of the crosslinker used, and should motivate the use of NDS methods to characterize SCNPs in the future. Monte Carlo simulations show that the distance between the pendant functional groups (*d*) and the randomness of the functional group distribution should both be considered for predicting *φ*_*λ*_. The simulation results for the PEG4 **A_2_** materials matched well with experimental values, but not for the PEG12 **A_2_** materials, which highlights the importance of the chemical dissimilarity between polymer **B_f_** and crosslinker **A_2_** when the length of crosslinker increases. Rheology studies suggest that while primary loops considerably impact the shear storage modulus (*G*′), higher-order loops and the distribution of pendant functional groups also play significant roles. Altogether, these results shed new light on the topology of one of the most widely used and studied classes of materials, and should guide the design of next generation sidechain-crosslinked networks and SCNPs.

## Conflicts of interest

The authors declare no competing financial interest.

## Supplementary Material

Supplementary information
