# Supplementary material for: Counting loops in sidechain-crosslinked polymers from elastic solids to single-chain nanoparticles
Source: Chem Sci. 2019 May 1;10(20):5332–7. doi: 10.1039/c9sc01297d (PMC6540913; doi:10.1039/c9sc01297d)
Supplement: Supplementary file 1 [file SC-010-C9SC01297D-s001.pdf]

Electronic Supplementary Material (ESI) for Chemical Science.
This journal is © The Royal Society of Chemistry 2019

*Supporting Information for:*

# Counting loops in sidechain-crosslinked polymers from elastic solids to single-chain nanoparticles

Junpeng Wang,† Rui Wang,‡ Yuwei Gu,† Alexandra Sourakov,† Bradley D. Olsen,‡* Jeremiah A. Johnson†*

†Department of Chemistry, Massachusetts Institute of Technology, Cambridge, MA 02139

‡Department of Chemical Engineering, Massachusetts Institute of Technology, Cambridge, MA 02139

## Table of Contents

General procedures. ........................................................................ 1

Quantifying the azide content in $\mathbf{B_{20H}}$ and $\mathbf{B_{20D}}$. ........................................ 7

Polymer network synthesis. ........................................................................ 9

Network degradation. ........................................................................ 10

Analysis of degradation products. ........................................................................ 10

Rheology. ........................................................................ 10

Monte Carlo Simulation Algorithm. ........................................................................ 12

References. ........................................................................ 14

## General procedures.

Unless otherwise specified, all reagents were purchased from Sigma-Aldrich and used without further purification. 6-azido-2,2-dimethylhexanoic acid and 6-azido-2,2-dimethylhexanoic-3,3,4,4,5,5,6,6-d8 acid were synthesized following previously reported procedures.[1] $\mathbf{A_2}$ with PEG4 or PEG12 were synthesized via modification of a reported procedure.[2] CuBr and $Me_6$TREN were purified according to previously reported procedures[1] and transferred into a $N_2$-filled glovebox before use.

Liquid chromatography–mass spectrometry (LC-MS) was performed on an Agilent 1260 LC system equipped with a Zorbax SB-C18 rapid resolution HT column and a Zorbax SB-C18 semi-preparative column. Solvent gradients consisted of mixtures of nano-pure $H_2O$ with 0.1% acetic acid (AcOH) and HPLC-grade acetonitrile (MeCN). Mass spectra were obtained using an Agilent 6130 single quadrupole mass spectrometer. $^1$H nuclear magnetic resonance ($^1$H NMR) spectra were recorded on Bruker AVANCE-400 NMR spectrometers in the Department of Chemistry Instrumentation Facility at MIT. Chemical shifts are expressed in parts per million (ppm); splitting patterns are designated as s (singlet), d (doublet), t (triplet), m (multiplet) and br (broad). Coupling constants J are reported in Hertz (Hz).

Gel permeation chromatography (GPC) was performed on an Agilent 1260 Infinity system equipped with three PL gel columns (103 Å, 104 Å, 105 Å) in series. Tetrahydrofuran (THF) was used as the mobile

phase. The system was calibrated using narrowly dispersed polystyrene standards. A sample concentration of 0.5 mg/mL was used.

Details of the rheology instrumentation and experiments are provided in the relevant section below.

## Synthesis of $B_{20H}$ and $B_{20D}$.

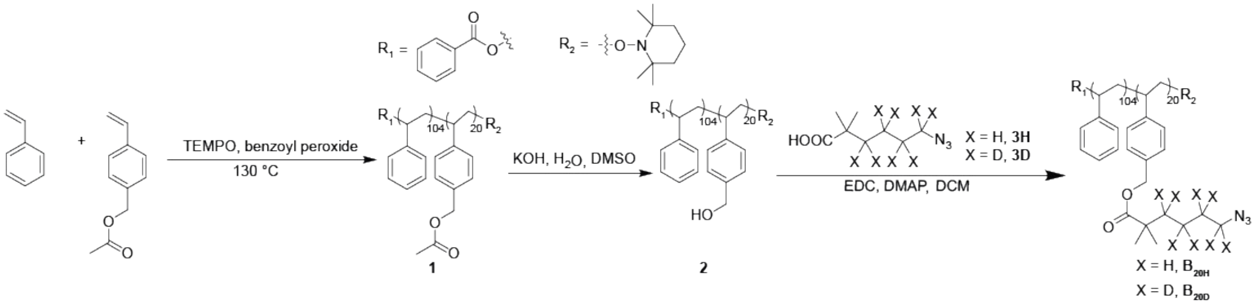


Synthesis of polymer **1**.

Styrene (3.0 g, 28.8 mmol), 4-vinyl benzyl acetate (0.92 g, 5.2 mmol), TEMPO (0.045 g, 0.288 mmol) and benzoyl peroxide (0.049 g, 0.202 mmol) were added to a 10 mL microwave reaction vial. The mixture was degassed with three freeze-pump-thaw cycles. The mixture was then heated to 95 °C and heated for 4 h under $N_2$ followed by additional stirring at 130 °C for 12 h under $N_2$. The polymerization was quenched in a liquid nitrogen bath. After warming, the crude reaction mixture was dissolved in 10 mL THF and added dropwise into methanol. The precipitated polymer was re-dissolved in 10 mL THF and re-precipitated via dropwise addition into methanol. The product was dried on a Schlenk line under vacuum at room temperature to provide a white powder (3.1 g, 79% yield). ($M_{n,\ GPC}$ = 14,600, $Đ$ = 1.16). $^1$H NMR (500 MHz, Chloroform-*d*) δ 7.23 – 6.86 (m, 17H), 6.83 – 6.21 (m, 13H), 5.02 (s, 2H), 2.40 – 1.01 (m, 25H).

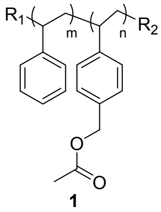


**1**

Determination of polymer composition:

$$\frac{m}{n} = \frac{17.60 + 12.18 - 4}{5} = 5.2, m + n = \frac{14{,}600}{104 \times \frac{5.2}{6.2} + 176 \times \frac{1}{6.2}} = 124, m = 104, n = 20.$$

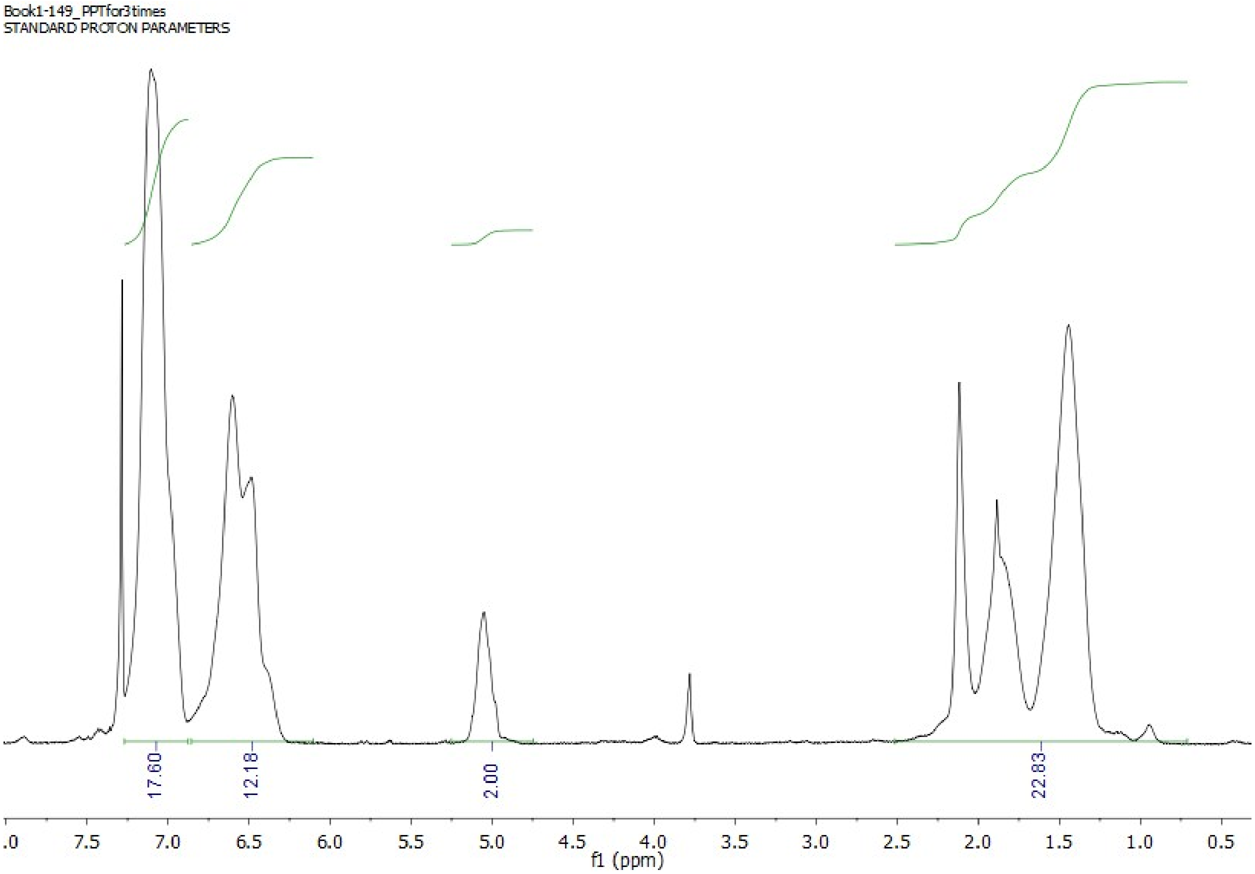


***Figure S1.*** $^1$H NMR (500 MHz, $CDCl_3$) spectrum of **1**.

Synthesis of polymer **2**.

To a 10 mL THF solution of polymer **1** (3.0 g) was added 10 mL 0.1 M KOH solution (5/95 H2O/DMSO). The reaction mixture was stirred for 4 h before being transferred to excess methanol. The resulting polymer precipitate was re-dissolved, re-precipitated in methanol, and dried on a Schlenk line overnight under vacuum, yielding **2** as a white powder (2.6 g, 98% yield). ($M_{n,\ GPC}$ = 13,200, $Đ$ = 1.16). $^1$H NMR (500 MHz, $CDCl_3$) δ 7.21 – 6.85 (m, 21H), 6.81 – 6.21 (m, 12H), 4.56 (s, 2H), 2.17 – 1.11 (m, 28H).

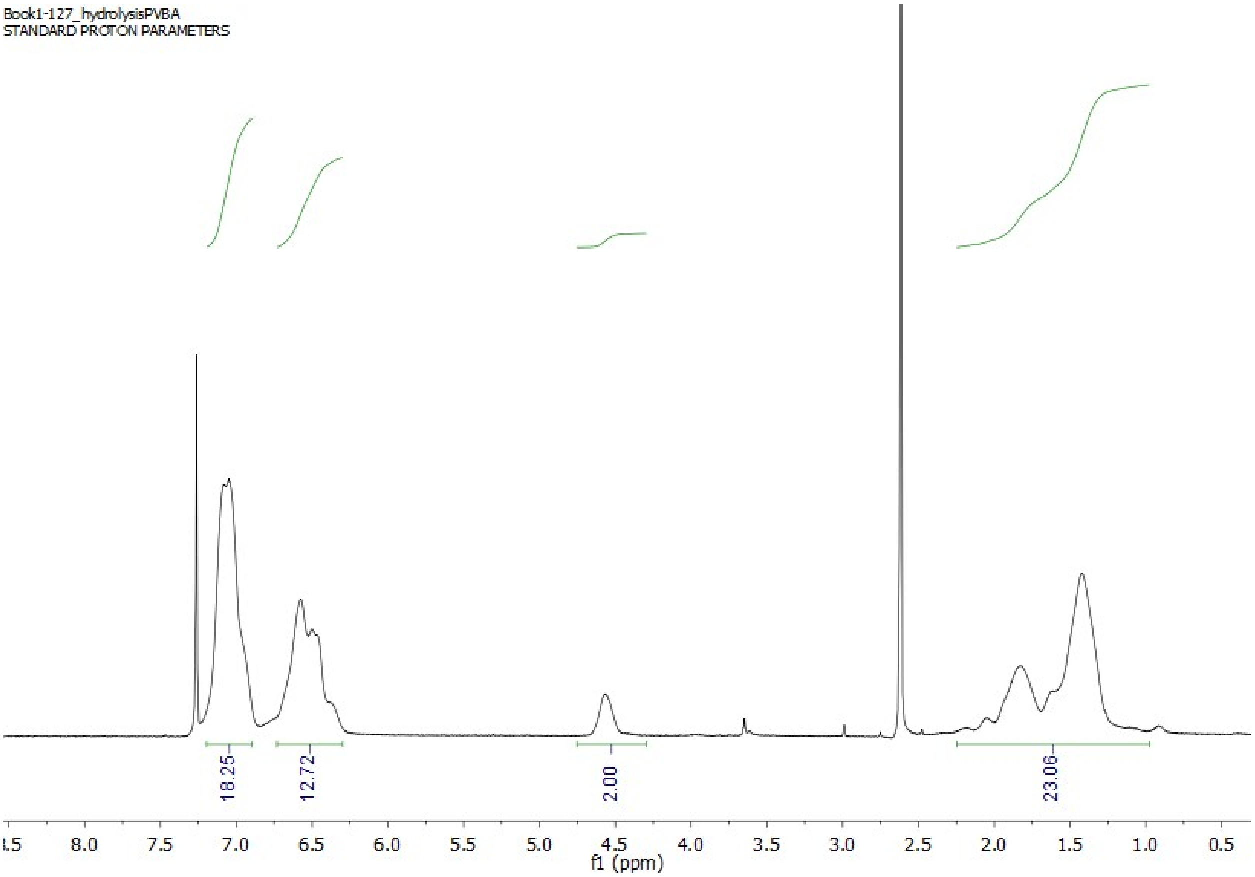


***Figure S2.*** $^1$H NMR (500 MHz, Chloroform-*d*) spectrum of polymer **2**.

Synthesis of $\mathbf{B_{20H}}$.

Polymer **2** (1 g, ~1.6 mmol -OH group) was dissolved in 10 mL $CH_2Cl_2$ in a round-bottom flask. To the solution was added 6-azido-2,2-dimethylhexanoic acid (0.59 g, 3.2 mmol, 2 eq), 4-dimethyl aminopyridine (0.098 g, 0.8 mmol, 0.5 eq), and (3-dimethylamino-propyl)-*N'*-ethylcarbodiimide hydrochloride (0.67 g, 3.52 mmol, 2.2 eq). The solution was stirred for 36 h. The reaction mixture was washed with water (2 × 10 mL) and brine (10 mL). The organic layer was added dropwise to methanol. The resulting polymer precipitate was re-dissolved in DCM, re-precipitated in methanol, and dried on a Schlenk line overnight under vacuum, yielding $\mathbf{B_{20H}}$ as a white powder (1.2 g, 93% yield). ($M_{n,\ GPC}$ = 17,300, $Đ$ = 1.17). $^1$H NMR (400 MHz, Chloroform-*d*) δ 7.21 – 6.82 (m, 19H), 6.78 – 6.19 (m, 11H), 5.02 (s, 2H), 3.30 – 2.98 (m, 2H), 2.36 – 0.81 (m, 32H).

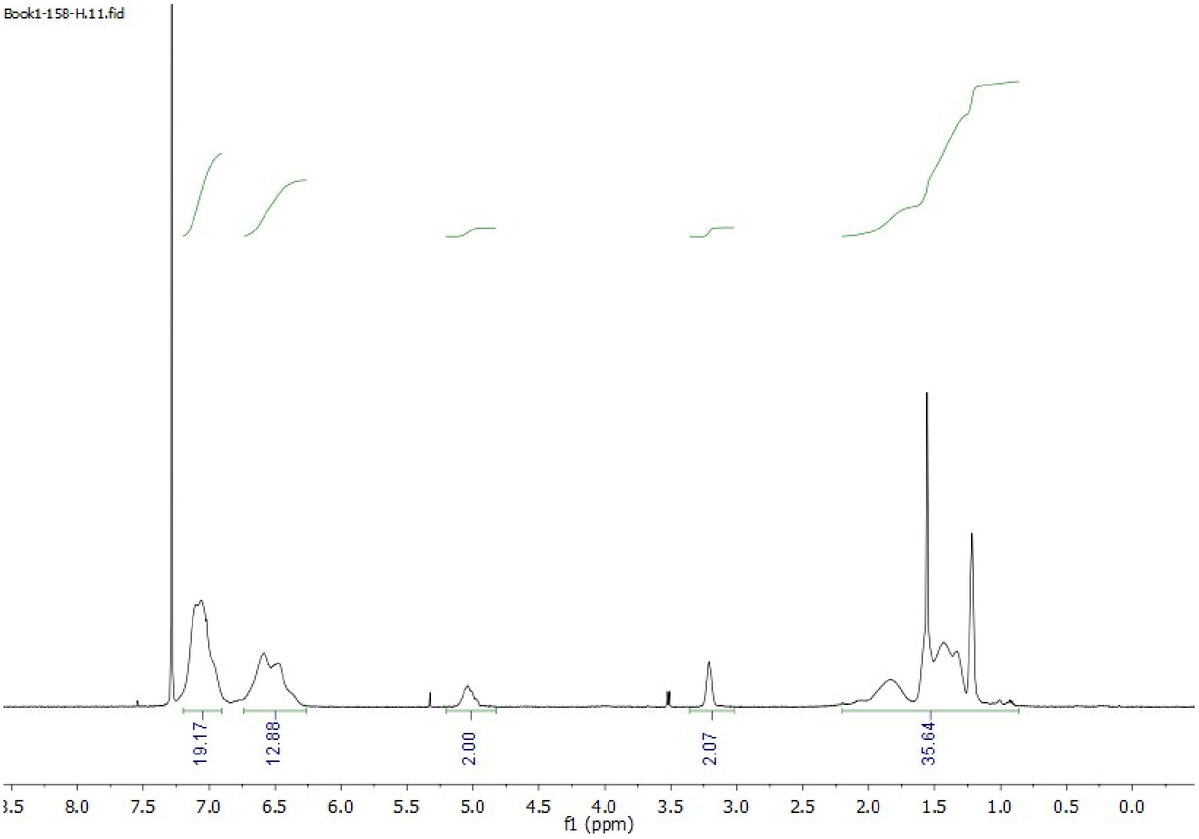


Figure S3. $^1$H NMR (400 MHz, Chloroform-*d*) of polymer $\mathbf{B_{20H}}$.

### $B_{20D}$

The synthesis of $\mathbf{B_{20D}}$ was conducted using similar procedure as that of $\mathbf{B_{20H}}$, except that 6-azido-2,2-dimethylhexanoic-3,3,4,4,5,5,6,6-d8 acid instead of the nonisotopic analogue was used. ($M_{n,\ GPC}$ = 17,300, $Đ$ = 1.17, calibrated by polystyrene standards). $^{1}H$ NMR (400 MHz, Chloroform-*d*) δ 7.04 (s, 18H), 6.51 (d, *J* = 47.9 Hz, 14H), 5.01 (s, 2H), 2.29 – 0.82 (m, 26H).

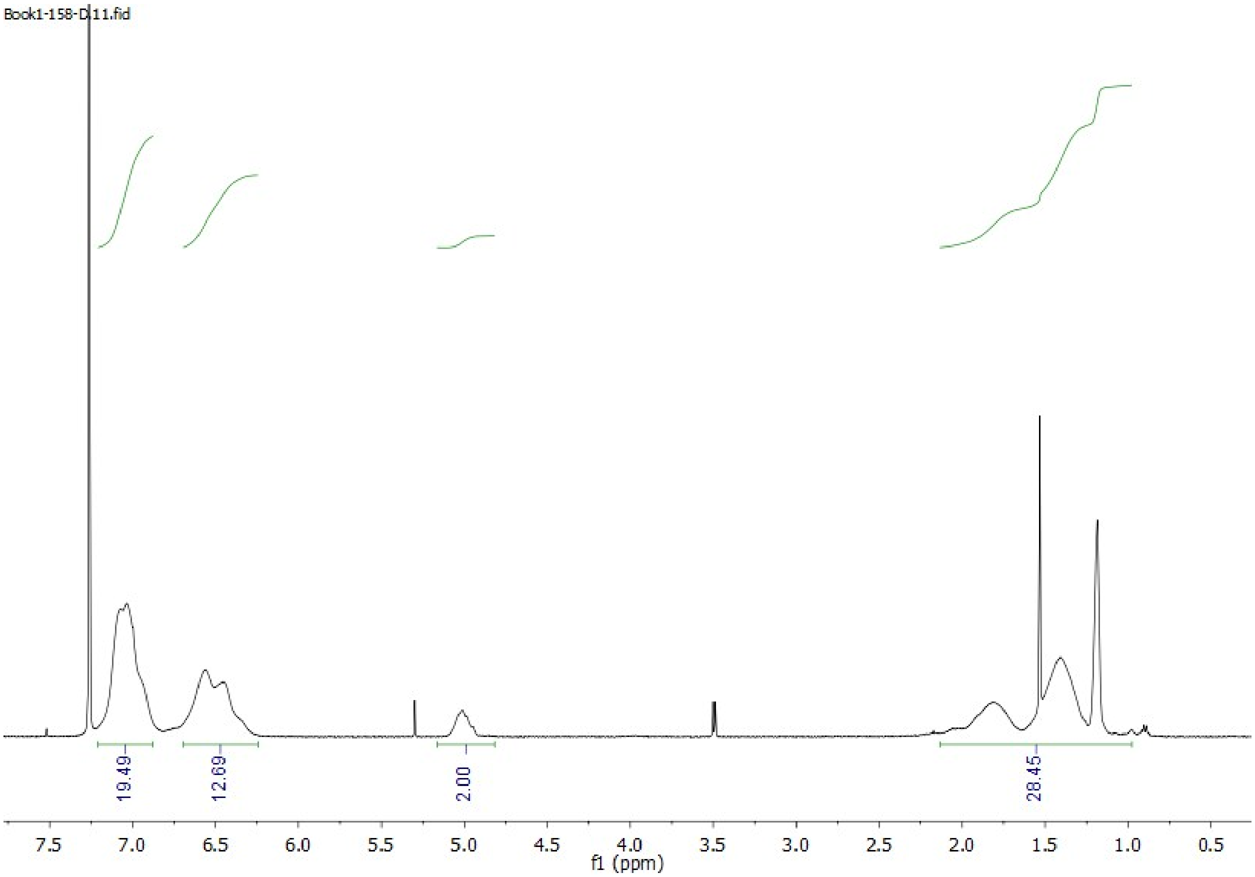


***Figure S4.*** $^{1}H$ NMR (400 MHz, $CDCl_3$) spectrum of polymer $\mathbf{B_{20D}}$.

## Quantifying the azide content in $B_{20H}$ and $B_{20D}$.

The azide ($-N_3$) content in $\mathbf{B_{20H}}$ and $\mathbf{B_{20D}}$ was quantified by reacting the polymers with a known but excess amount of propargyl 4-bromobenzamide by copper-catalyzed azide-alkyne cycloaddition (CuAAC). The detailed procedure is described as follows:

For $\mathbf{B_{20H}}$:

$\mathbf{B_{20H}}$ (4.67 mg) and propargyl 4-bromobenzamide (3.53 mg, 0.0148 mmol) were weighed and added into a vial (vial I). The mixture was dissolved in 250 µL of THF-$d_8$. In another vial, CuBr (1.4 mg, 0.01 mmol) and $Me_6$TREN (2.5 mg, 0.011 mmol) were dissolved in 250 µL of THF-$d_8$, and the resulting solution was added to vial I. The reaction was allowed to proceed overnight. After this time, the reaction mixture was characterized by $^1$H NMR.

The content of $-N_3$ in $\mathbf{B_{20H}}$ was determined according to: $\frac{\frac{1}{2.74} \times 0.0148\, mmol}{4.67\, mg} =$ 0.00116 mmol/mg.

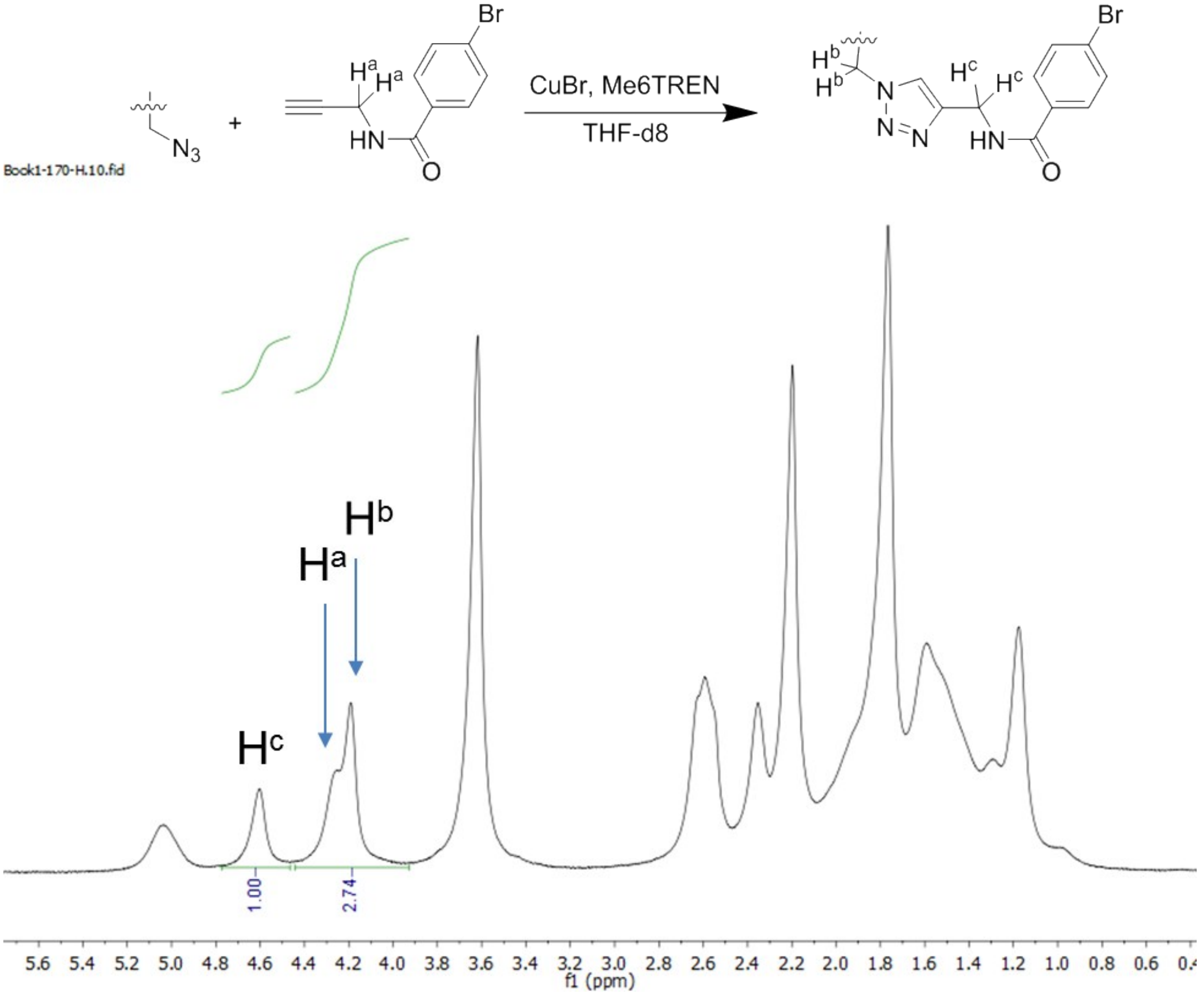


***Figure S5.*** $^1$H NMR (400 MHz, THF-$d_8$) spectrum of the reaction mixture used for quantification of the azide content of $\mathbf{B_{20H}}$.

For $\mathbf{B_{20D}}$:

$\mathbf{B_{20D}}$ (4.69 mg) and propargyl 4-bromobenzamide (3.29 mg, 0.0138 mmol) were weighed and added into a vial (vial I). The mixture was dissolved in 250 µL of THF-$d_8$. In another vial, CuBr (1.4 mg, 0.01 mmol) and Me6TREN (2.5 mg, 0.011 mmol) were dissolved in 250 µL of THF-$d_8$, and the solution was added to vial I. The reaction was allowed to proceed overnight. After this time, the reaction mixture was characterized by $^{1}$H NMR.

The content of $-N_3$ in $\mathbf{B_{20H}}$ was determined according to: $\dfrac{\frac{1}{1+1.57}\times 0.0138\,mmol}{4.69\,mg} =$ 0.00114 mmol/mg.

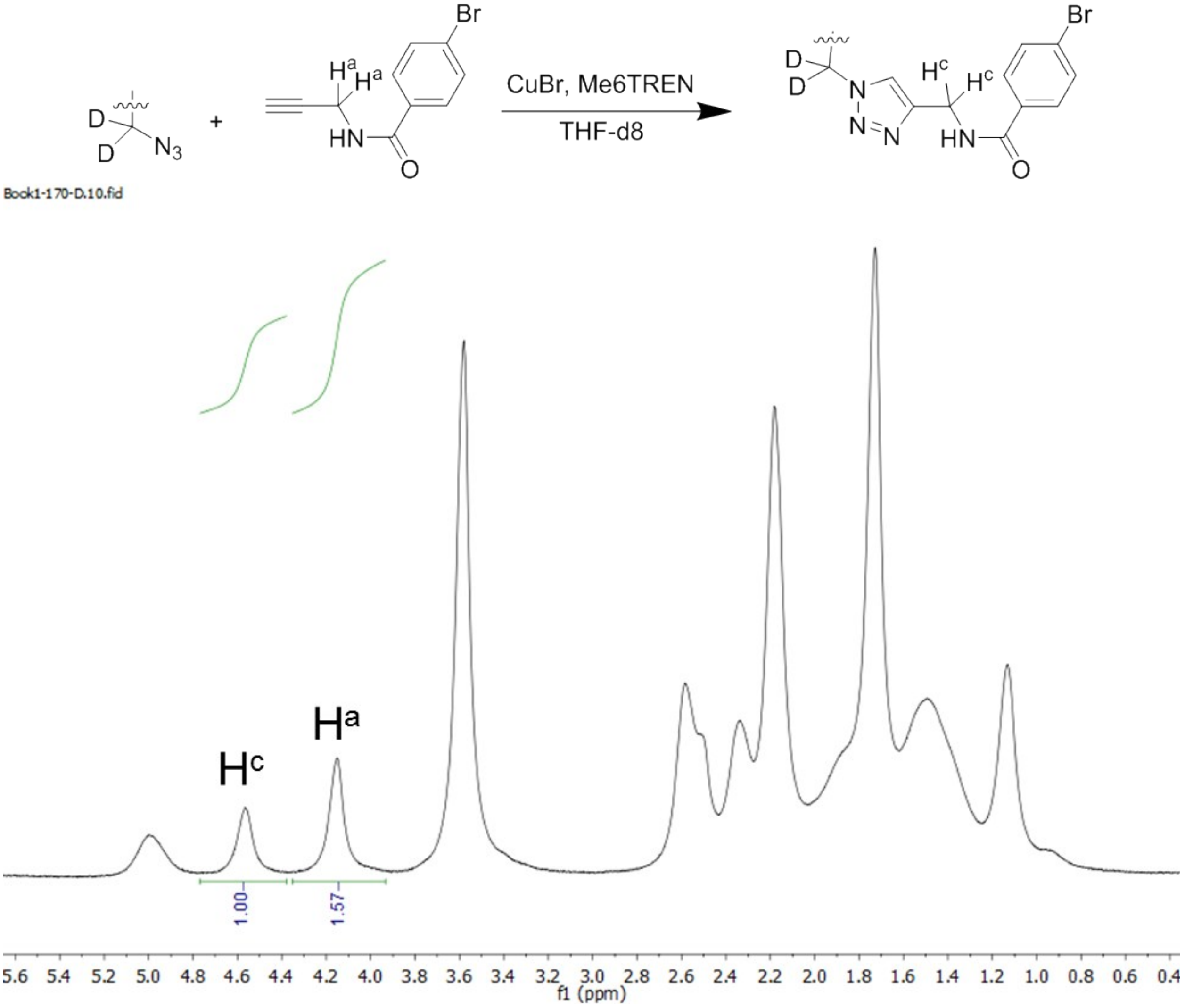


***Figure S6.*** $^{1}$H NMR (400 MHz, THF-$d_8$) spectrum of the reaction mixture used for quantification of the azide content of $\mathbf{B_{20D}}$.

## Polymer network synthesis.

**$A_2$**, **$B_{20H}$**, and **$B_{20D}$** were weighed in a 20 mL scintillation vial and transferred into a $N_2$ glovebox. All of the following procedures were carried out in the same $N_2$ glovebox. A stock solution of the polymers and the desired crosslinker (80 mM **$A_2$**, **$B_{20H}$** with 80 mM -$N_3$ and **$B_{20D}$** with 80 mM –$N_3$) was prepared in a volumetric flask using *N*-methyl-2-pyrrolidone (NMP) solvent. A stock solution of the mixture of CuBr and $Me_6$TREN (400 mM CuBr, 440 mM $Me_6$TREN) in NMP was prepared in another vial. Polymer networks were formed by sequentially adding NMP, polymer and crosslinker solution, and CuBr/$Me_6$TREN solution using a micropipette into 2 mL scintillation vials with white urethane solid caps. Note: In most cases, 1 equivalent of CuBr and 1.1 equivalent of $Me_6$TREN were used. In order to study the impact of kinetics on the loop fraction of the **$A_2$** PEG12 network, 0.2 equivalents of CuBr of 0.22 equivalents of $Me_6$TREN were used and the results were compared to the 1 equivalent version, see Figure S6. Upon mixing of the reaction components, the vial was held on a vortex mixer for 30 s to ensure homogeneous mixing. Different polymer concentrations were obtained by changing the amount of NMP, polymer and crosslinker solution, and CuBr/$Me_6$TREN solution, and 3 samples were made for each concentration. The samples were allowed to react for 24 h to achieve complete conversion.

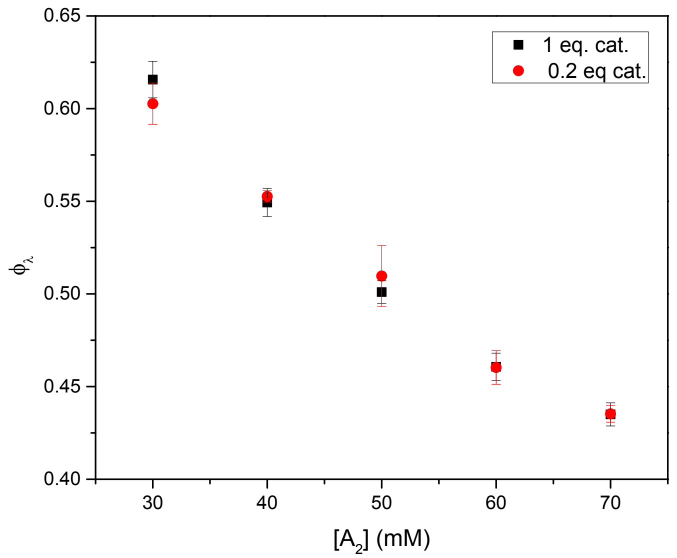


***Figure S7.*** Measured primary loop fractions $\phi_\lambda$ for different catalyst loadings (1 equivalent: black square; 0.2 equivalents: red dot) at different initial **$A_2$** PEG12 concentrations.

**Network degradation.**
The samples were removed from the glovebox and were exposed to 0.1 M KOH (5/95 $H_2O$/DMSO) while vortexing. The samples were allowed to react for ~4 h prior to analysis by LC-MS.

**Analysis of degradation products.**
The degradation products were analyzed by LC-MS using the Single Ion Mode (SIM) feature. The principal isotope peak for each degradation product was extracted in the "Extract Ion" feature of ChemStation, and quantified using the integration feature for each extracted ion.

**Rheology.**
Gel samples (300 µL in total) for rheology were prepared in NMP in 4 mL vials. The vials were broken with a hammer to remove the gels, which were subsequently cut with an 8 mm hole puncher (purchased from McMaster-Carr) and transferred onto the rheometer. Rheology experiments were performed at 25 °C on a TA Instruments Discovery Hybrid Rheometer HR-2 rheometer with an 8 mm standard parallel steel plate geometry. Evaporation of NMP was negligible within the typical measurement time (< 20 minutes). A strain sweep was performed to determine the linear viscoelastic region for frequency sweep studies. A frequency sweep was then performed from 0.1 Hz to 10 Hz at a constant strain of 0.5%. The reported shear storage modulus $G'$ was determined at 1 rad/s. full frequency sweep curves are shown in Figures S7-S8.

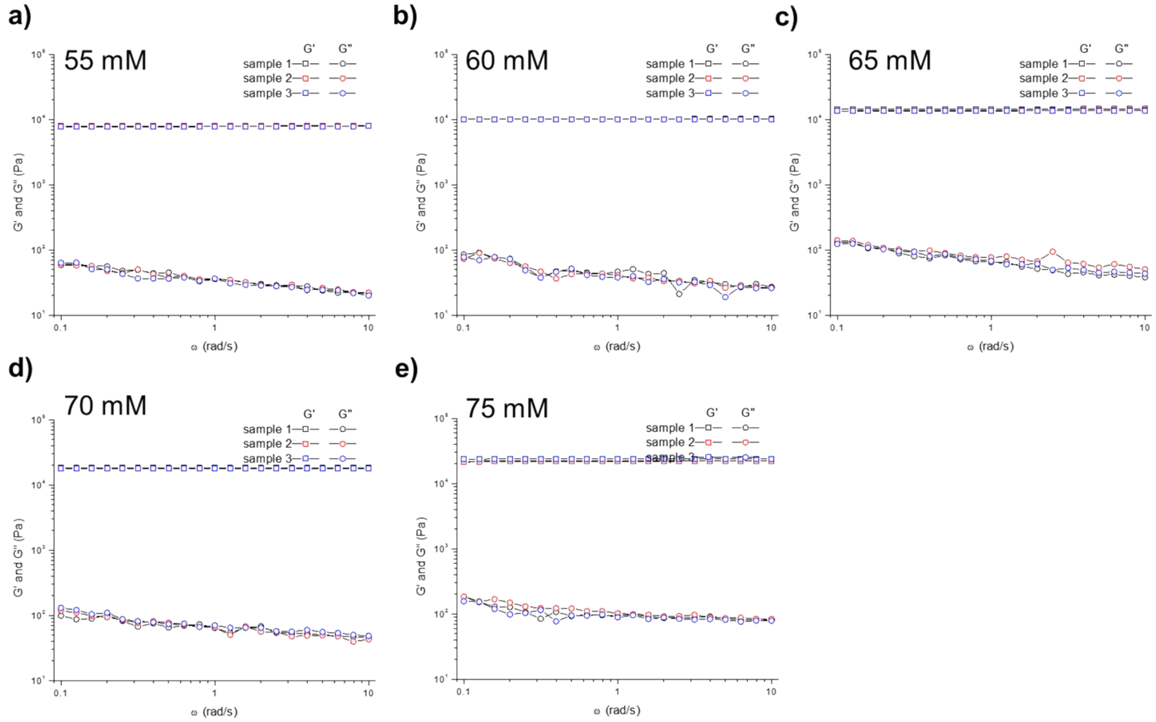


***Figure S8.*** Frequency sweep rheological data for $\mathbf{A_2}$ PEG4 gels with different initial concentrations of $\mathbf{A_2}$ crosslinker: (a) 55 mM; (b) 60 mM; (c) 65 mM; (d) 70 mM; (e) 75 mM.

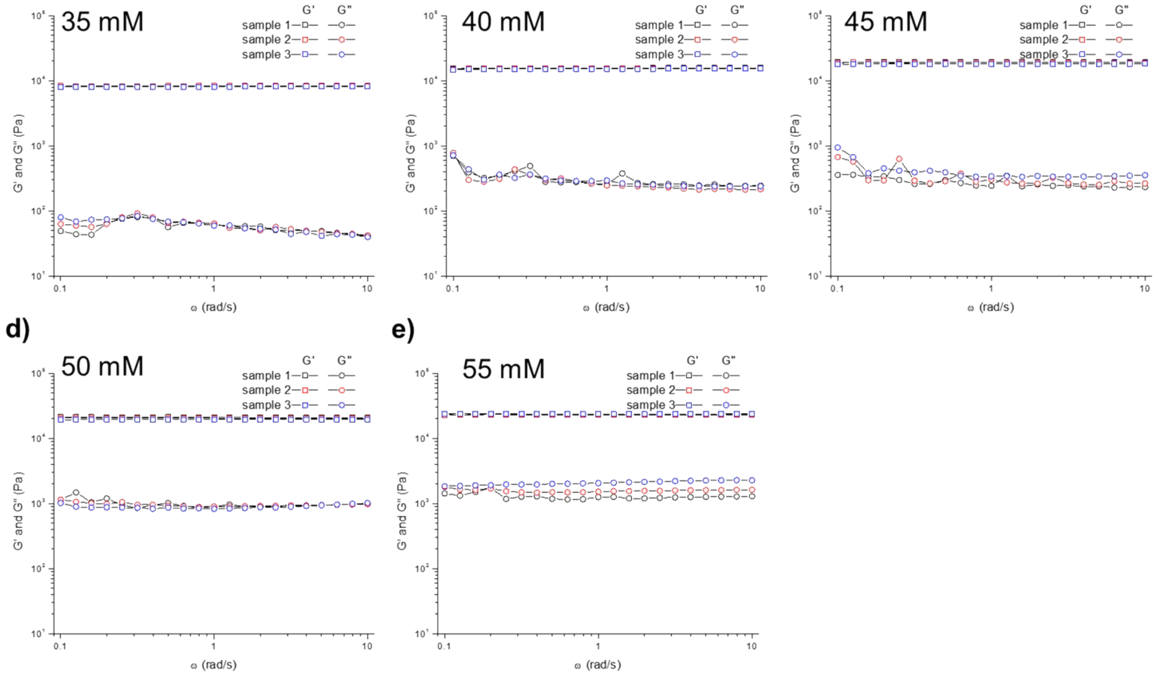


***Figure S9.*** Rheological data for $\mathbf{A_2}$ PEG12 gels with different initial concentrations of $\mathbf{A_2}$ crosslinker: (a) 35 mM; (b) 40 mM; (c) 45 mM; (d) 50 mM; (e) 55 mM.

## Monte Carlo Simulation Algorithm.

The kinetic Monte Carlo simulation algorithm previously developed for polymer networks synthesized via end-linking of bifunctional polymers and point-like multifunctional crosslinkers[3] is modified to count the 1° loops in side-chain crosslinked polymer networks. The polymer networks considered are formed via crosslinking bifunctional polymers (**$A_2$**) and multifunctional macromolecules (**$B_f$**). Here we assume that the crosslinking process is not diffusion-limited but reaction-limited, which allows simulation from a purely topological perspective: the spatial information of the polymers and junctions can be ignored, and the simulation can be simplified by only tracking the topological distance between reactive groups. Based on the polymer concentration $c$, $N_A$ **$A_2$** polymers and $N_B$ **$B_f$** junctions are initially placed in a system of volume $V$ ($c = N_A / V$). **$A_2$** is modeled as a uniform flexible Gaussian chain with degree of polymerization *N* and Kuhn length *b*. To explore how the distribution of groups on **$B_f$** affects 1° loop formation, three conditions were explored: centralized functional groups (equivalent to previous studies of loops in gelation using point crosslinkers[3]), functional groups evenly spaced by *d* monomers, and randomly distributed functional groups. Networks are constructed by sequentially selecting a pair of unreacted **A-B** groups at each step with a probability $P_{AB}$ reflecting the topological connection between these two groups,

$$P_{AB} = \frac{\frac{1}{V} + \left( \frac{3}{2\pi R_{AB}^2} \right)^{3/2}}{\sum_{ij} \left[ \frac{1}{V} + \left( \frac{3}{2\pi R_{ij}^2} \right)^{3/2} \right]} \tag{S1}$$

where the summations are taken over all unreacted **A-B** pairs. $R_{AB}^2 = n_A b_A^2 + n_B b_B^2$ is the square end-to-end distance between the selected pair of unreacted **A-B** groups, where $n_A$ and $n_B$ are the number of **A** and **B** Kuhn monomers, respectively. $b_A$ and $b_B$ are the Kuhn lengths of the **A** monomer and **B** monomer, respectively. The distance between unreacted **A-B** pairs are updated after the selected *A*-*B* pair reacts. If a pair of *A* and *B* functional groups reacts, their distance is set to be 0. This reaction creates a new pathway between all groups that are connected to the selected *A* and *B* groups. If this new pathway shortens the

topological distance $R_{ij}$ will be updated, which then requires reevaluating the denominator of the selection probability in Eq. S1.

The calculation of $R_{ij}$ depends on the distribution of reactive groups on $\mathbf{B}_f$. For centralized functional groups (equivalent to point crosslinkers), the distance between any two **B** groups belonging to the same $\mathbf{B}_f$ is 0. Therefore, $R_{AB}^2 = n_A b_A^2$, where **B** monomers do not contribute to the end-to-end distance. For evenly spaced functional groups, $n_B$=5 for any two adjacent **B** groups belonging to the same $\mathbf{B}_f$, which is based on the DP of unfunctionalized PS = 100 and degree of functionality = 20 in $\mathbf{B_{20H}}$ and $\mathbf{B_{20D}}$. The distance between two nonadjacent **B** groups belonging to the same $\mathbf{B}_f$ can thus be calculated straightforwardly.

For the case of randomly distributed functional groups, a separate Monte Carlo simulation is used to generate the distribution of crosslinkers along the $\mathbf{B}_f$ macromolecules. 1000 $\mathbf{B}_f$ macromolecules are grown sequentially at the same time. At each step, the length each $\mathbf{B}_f$ macromolecule grows (or equivalently the number of **B** monomers that can join to the chain to separate two functionalized **B** monomers) has the following probability,

$$P(n) = (1-F)^n F \tag{S2}$$

where $F$ is the fraction of functionalized **B** monomers in the entire $\mathbf{B}_f$ macromolecule. For DP of unfunctionalized PS = 100 and degree of functionality = 20 in $\mathbf{B_{20H}}$ and $\mathbf{B_{20D}}$, $F$ =20/120. The average distance between functionalized **B** monomers $\langle n \rangle = (1-F)/F = 5$, which is consistent with the value of evenly spaced crosslinkers. Unlike the case of evenly spaced crosslinkers, the distance between two adjacent functionalized **B** monomers belonging to the same $\mathbf{B}_f$ macromolecule has a distribution, which will change the probability of loop formation as shown in Eq. S1. The distances between any two adjacent functionalized **B** monomers belonging to the same $\mathbf{B}_f$ are recorded, and the distances between two nonadjacent **B** groups can be straightforwardly calculated.

In the simulation of loop formation, 10000 $\mathbf{A_2}$ bifunctional polymers and 1000 $\mathbf{B_{20}}$ macromolecules are initially placed in the system at various concentrations. The number of molecules is chosen to be large enough so that the loop fractions are not sensitive to the system size. Ten parallel simulations were run for

each set of parameters to obtain the average fraction of 1° loops. In the simulation, intrinsic parameters of PEG (Kuhn length $b_A$ =1.1 nm and molar mass of Kuhn monomer $m_A$ =137 g/mol) are used based on reported values.[4] For PS, Kuhn length $b_B$ =1.8nm and molar mass of Kuhn monomer $m_A$ =5000 g/mol are used according to measurements of the static and dynamic properties of PS solutions.[5]

## References.

1. K. Kawamoto, M. Zhong, R. Wang, B. D. Olsen and J. A. Johnson, Loops versus branch functionality in model click hydrogels, *Macromolecules*, 2015, **48**, 8980-8988.

2. M. Shan, A. Bujotzek, F. Abendroth, A. Wellner, R. Gust, O. Seitz, M. Weber and R. Haag, Conformational analysis of bivalent estrogen receptor ligands: from intramolecular to intermolecular binding, *ChemBioChem*, 2011, **12**, 2587-2598.

3. R. Wang, A. Alexander-Katz, J. A. Johnson and B. D. Olsen, Universal cyclic topology in polymer networks, *Phys. Rev. Lett.*, 2016, **116**, 188302-1-5.

4. M. Rubinstein and R. H. Colby, *Polymer Physics* (Oxford University Press, Oxford, 2003).

5. Y. Ding and A. P. Sokolov, Comment on the dynamic bead size and Kuhn segment length in polymers: example of polystyrene, *J. Polym. Sci., Part B: Polym. Chem.*, 2004, **42**, 3505-3511.
